# Protective Effect of Tat PTD-Hsp27 Fusion Protein on Tau Hyperphosphorylation Induced by Okadaic Acid in the Human Neuroblastoma Cell Line SH-SY5Y

**DOI:** 10.1007/s10571-015-0199-1

**Published:** 2015-05-20

**Authors:** Sunghyun Choi, Jae Hoon Oh, Hyeseon Kim, So Hee Nam, Jeehae Shin, Jong-Sang Park

**Affiliations:** Department of Chemistry, Seoul National University, 599 Gwanak-ro, Gwanak-gu, Seoul, 151-747 Republic of Korea

**Keywords:** Tau protein, Hyperphosphoylation, Alzheimer’s disease, Heat shock protein 27, Protein transduction domain

## Abstract

Alzheimer’s disease (AD) is an age-related disorder that causes a loss of brain function. Hyperphosphorylation of tau and the subsequent formation of intracellular neurofibrillary tangles (NFTs) are implicated in the pathogenesis of AD. Hyperphosphorylated tau accumulates into insoluble paired helical filaments that aggregate into NFTs; therefore, regulation of tau phosphorylation represents an important treatment approach for AD. Heat shock protein 27 (Hsp27) plays a specific role in human neurodegenerative diseases; however, few studies have examined its therapeutic effect. In this study, we induced tau hyperphosphorylation using okadaic acid, which is a protein phosphatase inhibitor, and generated a fusion protein of Hsp27 and the protein transduction domain of the HIV Tat protein (Tat-Hsp27) to enhance the delivery of Hsp27. We treated Tat-Hsp27 to SH-SY5Y neuroblastoma cells for 2 h; the transduction level was proportional to the Tat-hsp27 concentration. Additionally, Tat-Hsp27 reduced the level of hyperphosphorylated tau and protected cells from apoptotic cell death caused by abnormal tau aggregates. These results reveal that Hsp27 represents a valuable protein therapeutic for AD.

**Electronic supplementary material:**

The online version of this article (doi:10.1007/s10571-015-0199-1) contains supplementary material, which is available to authorized users.

## Introduction

Alzheimer’s disease (AD) is a progressive neurological disorder that causes memory loss. Various pathological hallmarks of AD include synaptic and neuronal loss, amyloid plaques primarily composed of the 42-residue hydrophobic β-amyloid peptide (Aβ) (Selkoe 2002), and neurofibrillary tangles (NFTs) composed of aggregates of hyperphosphorylated tau, which is a microtubule-associated protein (Kosik and Shimura 2005). Amyloid plaques and NFTs are considered as the primary factors involved in the pathogenesis of AD (Medeiros et al. 2010). Although previous studies have primarily focused on the role of β-amyloid peptides, recent studies on the role of tau in AD pathogenesis have indicated that hyperphosphorylated tau aggregates into insoluble paired helical filaments (PHFs), which induce neuronal dysfunction (Santa-Maria et al. 2012). Moreover, these two pathological hallmarks demonstrate synergistic effects on synaptic dysfunction (Crimins et al. 2013; Chabrier et al. 2014).

As a microtubule-associated protein, aggregates of normal tau protect cells against toxic hyperphosphorylation, although neuronal death occurred following a period of survival (Hernandez and Avila 2007). However, tau is not only abnormally phosphorylated but also aggregates into insoluble forms such as PHFs and NFTs (Andorfer et al. 2003; Citron 2010) in various AD mouse models (Götz et al. 2001; Denk and RichardWade-Martins 2009; Baglietto-Vargas et al. 2014). Therefore, an approach that reduces the level of hyperphosphorylated tau would represent a valuable treatment for AD (Buée et al. 2000). Tau can bind heat shock proteins (HSPs), which trigger the recruitment of CHIP (which is a co-chaperone that exhibits E3 activity), to the complex. CHIP has been shown to interact with tau, appears to work in concert with both Hsp70 and Hsp90 in degrading toxic tau species (Saidi et al. 2015). CHIP induces the ubiquitination of tau and activates its degradation when tau is defective (Salminen et al. 2011). In addition, The BAG2/Hsp70 complex is tethered to the microtubule and this complex can capture and deliver Tau to the proteasome for ubiquitin-independent degradation (Carrettiro et al. 2009). Curcumin also downregulated the levels of phosphorylated tau, which may be potentially attributed to the curcumin-induced upregulation in BAG2 levels in the neurons (Patil et al. 2013). Hsp27 directly associates with hyperphosphorylated tau or PHFs and regulates cell survival by eliminating tau aggregates (Shimura et al. 2004b).

Heat shock proteins are induced in response to cellular stress as molecular chaperones that inhibit protein aggregation (Read and Gorman 2009). HSPs can prevent apoptosis and increase cell viability during cellular stress (Shimura et al. 2004a; Kwon et al. 2007). HSPs are also critical regulators of normal neural physiological function and cell stress responses (Stetler et al. 2010; Arrigo 2013; Song et al. 2014). However, the therapeutic effects of exogenous Hsp27 on disease models have not been investigated. Therefore, in this study, we investigated whether Hsp27 could reduce hyperphosphorylated tau in AD-induced SH-SY5Y cells.

To more effectively deliver Hsp27 into cells, we combined the HIV protein transduction domain (PTD) Tat with the Hsp27 protein (Tat-Hsp27). HIV Tat (11 residues, YGRKKRRQRRR) can rapidly transduce into cells (Becker-Hapak et al. 2001) and deliver full-length proteins into cells (Green et al. 2003). Tat-Hsp27 effectively reduced the phosphorylation of tau and rescued the cell death caused by abnormal tau aggregates. Therefore, our study suggests that Tat-Hsp27 may represent a potential protein therapeutic for tau-induced neurodegeneration.

## Materials and Methods

### Construction of the Expression Vector

We designed a bacterial expression vector (His6-Tat-Hsp27) containing hexahistidine leader sequence, 11-amino acid Tat PTD sequence (YGRKKRRQRRR), and Hsp27 protein sequence. The Hsp27 fragment (BD Bioscience) was generated by PCR using human cDNA as a template and oligonucleotide primers containing *Bam*HI and *Xho*I restriction sites. The pET-28a vector (Novagen) and the Tat PTD were digested with *Nde*I and *Bam*HI and ligated. The ligated vector was transformed into chemically competent DH5α *E. coli* cells (Enzynomics, Korea). After purification of the plasmid containing the Tat PTD, the plasmid and the Hsp27 fragment were digested with *Bam*HI and *Xho*I and ligated. We also designed pET28a vector containing Hsp27 (wt-Hsp27) genetically fused with a hexahistidine (His6) tag. The plasmid was constructed by digesting the pET28a vector with *BamHI* and *XhoI* restriction endonucleases and ligating the Hsp27 fragment digested with the same restriction enzymes into the cut vector. The recombinant plasmids (pET28a-Tat-Hsp27, pET28a-Hsp27) were transformed into the Escherichia coli strain BL21 (DE3) for protein expression (Enzynomics, Korea).

### Expression and Purification of Hsp27

The recombinant plasmid (His6-Tat-Hsp27) was transformed into the *E. coli* strain BL21(DE3) for protein expression. Transformed cells were plated on an LB agar (Merck) plate containing kanamycin (Sigma-Aldrich) and incubated overnight at 37 °C. Two-hundred milliliters of LB medium containing 1 mM kanamycin was inoculated with a single colony and incubated overnight at 37 °C with shaking at 200 rpm. The following day, 1 L of LB medium was inoculated with this preculture and incubated at 37 °C until an OD_600_ of 0.5 was reached. Protein expression was induced by the addition of Isopropyl β-D-1-thiogalactopyranoside (MB cell, Korea) at a final concentration of 1 mM, and the cells were incubated at 37 °C for an additional 4 h. The cells were centrifuged at 6000 rpm for 15 min at 4 °C and resuspended in binding buffer (20 mM Tris–HCl, 500 mM NaCl, 35 mM imidazole, pH 7.5). Cells were lysed by sonication on ice using a sonicator (Sonics Vibra-Cell VCX 750, Sonic & Materials Inc., USA) with 1-s pulses and 8-s pauses for 30 min. After sonication, the lysates were centrifuged at 10,000 rpm for 15 min. The clarified lysate was loaded onto a pre-equilibrated HisTrap HP column (GE Healthcare). His-tagged Tat-Hsp27 protein was eluted with elution buffer (20 mM Tris–HCl, 500 mM NaCl, 1 M imidazole, pH 7.5). His-tagged Tat-Hsp27 was further purified using size exclusion chromatography on a HiLoad 16/600 Superdex 200 prep grade column (GE Healthcare) using 20 mM Tris–HCl (pH 7.5) and 100 mM NaCl. Wt-Hsp27 purification was performed in the same manner as in the purification step above. For the detection of Tat-Hsp27 delivery into cells, we conjugated FITC to Tat-Hsp27 using an FITC labeling kit (Thermo Scientific). The concentration of protein was determined using BCA protein assay kit (Pierce).

### Labeling of wt-Hsp27, TAT-Hsp27

To eliminate any primary amines or ammonium ions from previous buffer, the buffers for both wt-Hsp27 and TAT-Hsp27 were exchanged with 50 mM sodium borate of pH 8.5 using Amicon ultra centrifugal filters. Two-hundred thirty micrograms of the prepared protein was added to a vial of FITC Reagent (50 μg). The reaction mixture was incubated for 60 min at room temperature protected from light. After adding the mixtures to the spin columns, they were centrifuged to remove any excess FITC at 1000×*g* for 1 min.

### Cell Culture

SH-SY5Y human neuroblastoma cells were grown in Roswell Park Memorial Institute 1640 medium supplemented with 10 % fetal bovine serum and 1 % antibiotic–antimycotic under an atmosphere of 5 % CO_2_ and 95 % air (all from WELGENE). The medium was refreshed every three days. Cells below passage 24 were used for experiments. We chose the SH-SY5Y cell line because these human neuroblastoma cells express constantly endogenous tau.

### Western Blot Analysis

For Western blot analysis, the cells treated with Tat-Hsp27 and okadaic acid were washed in DPBS and harvested in RIPA buffer (Pierce). To prevent from further phosphorylation, we added Protease inhibitor cocktail and phosphatase inhibitor cocktail composed of sodium orthovanadate, sodium molybdate, sodium tartrate, and imidazole (both from Sigma-Aldrich) into the cell lysates. Lysates were then centrifuged at 15,000×*g* for 15 min at 4 °C. The supernatant was separated in a 12 % SDS-PAGE gel, and the proteins were transferred to a nitrocellulose membrane. After blocking in 5 % skim milk/TBST (Tris-buffered saline and 0.1 % Tween 20, pH 7.5), the membrane was washed in TBST three times (15 min each) and incubated with primary antibody overnight at 4 °C. The membrane was subsequently washed and incubated with the appropriate secondary antibody (HRP-conjugated goat anti-mouse and goat anti-rabbit) for 2 h at room temperature. Protein signals were developed with ECL Western Blotting Detection Reagent (Amersham Pharmacia Biotech, Piscataway, NJ, USA) exposed to X-ray film. Band intensities were calculated with the *Image J* software, and the protein band of interest was normalized to β-actin. Then, tau hyperphosphorylation was normalized to the total tau as a ratio, pTau/Total tau. Peroxidase-linked anti-rabbit and anti-mouse IgGs were purchased from Santa Cruz Biotechnology (USA). Rabbit polyclonal anti-tau phosphoserine 199/202 antibody and mouse monoclonal anti-tau-1 antibody were obtained from Millipore (USA). Anti-human total tau monoclonal antibody (HT 7), rabbit anti-Hsp27 polyclonal antibody, and anti Phospho-PHF-tau (detected phospho-S202, -T205) monoclonal antibody (AT 8) were purchased from Pierce Biotechnology (USA). Anti-HIV1 tat monoclonal antibody was purchased from Abcam (USA). Synthesized tat peptides (YGRKKRRQRRR) were purchased from PEPTRON (Korea).

### Immunocytochemistry

The SH-SY5Y cells were seeded on 2-well slides (Lab-Tek chamber, Nalge Nunc, NY) at a density of 5.0 × 10^5^ per well, treated with 2 μM Tat-Hsp27 for 2 h, and then treated with 10 nM okadaic acid for 14 h. The cells were rinsed two times with PBS and fixed with 4 % paraformaldehyde for 30 min. The cells were subsequently permeabilized for 10 min with 0.5 % Triton X-100 followed by three 5-min washes in PBS. To reduce nonspecific binding, we used PBS containing 0.5 % bovine serum albumin as blocking buffer. After incubation with the blocking buffer for 1 h, the cells were incubated with the primary antibody AT8 (anti Phospho-PHF-tau detected phospho-S202, -T205) for 4 h, followed by three 5-min washes. The cells were subsequently incubated with goat anti-rabbit (H + L) FITC-conjugated antibody. After washing with PBS, cells were imaged using an image restoration microscope (Applied Precision, USA).

### Cell Viability/Cytotoxicity Assay

#### A Cytotoxicity Assay was Performed

Using a Cell Counting Kit-8 (Dojindo, Korea). SH-SY5Y cells were plated in a 96-well plate at 5.0 × 10^4^ in 100 μL of RPMI 1640 medium containing 10 % FBS. After incubation for 48 h, cells were treated with 0.5 μM or 2 μM Tat-Hsp27 for 2 h. Subsequently, 10nM and 50nM okadaic acid were added for 6 h to induce abnormally phosphorylated tau. Ten microliters of CCK-8 solution was added, and the cells were incubated at 37 °C for 2 h. The absorbance at 450 nm was measured using a microplate reader (Molecular Devices Co., Menlo Park, CA).

### In Situ Terminal Deoxynucleotidyl Transferase dUTP Nick-End Labeling (TUNEL) Assay

SH-SY5Y cells were seeded on a 2-well slide at a density of 5.0 × 10^5^ per well, treated with 2 μM Tat-Hsp27 for 2 h, and then treated with 10 nM okadaic acid for 14 h. The cells were fixed by immersion in PBS containing 4 % formaldehyde (pH 7.4). After washing with PBS, the cells were permeabilized in 0.2 % Triton X-100 and washed again. After treatment with equilibration buffer, the cells were added to rTdT incubation buffer containing nucleotide mix. To terminate the reaction, saline sodium citrate buffer was added to the cells; nuclei were stained with DAPI and the mounting media used was Vectashield. The cells were analyzed under a fluorescence microscope at 520 nm (green fluorescence) and 460 nm (blue, DAPI).

### Statistical Analysis

Statistical analysis was performed with the Statistical Package for the Social Sciences (SPSS, IBM Corporation, NY, USA). Statistical significance was determined by analysis of variance (ANOVA) with Tukey’s HSD test and student’s *t* test. All results are presented as the mean ± standard deviation, and statistical significance was considered at the 5 % level.

## Results

**Fig. 1:**
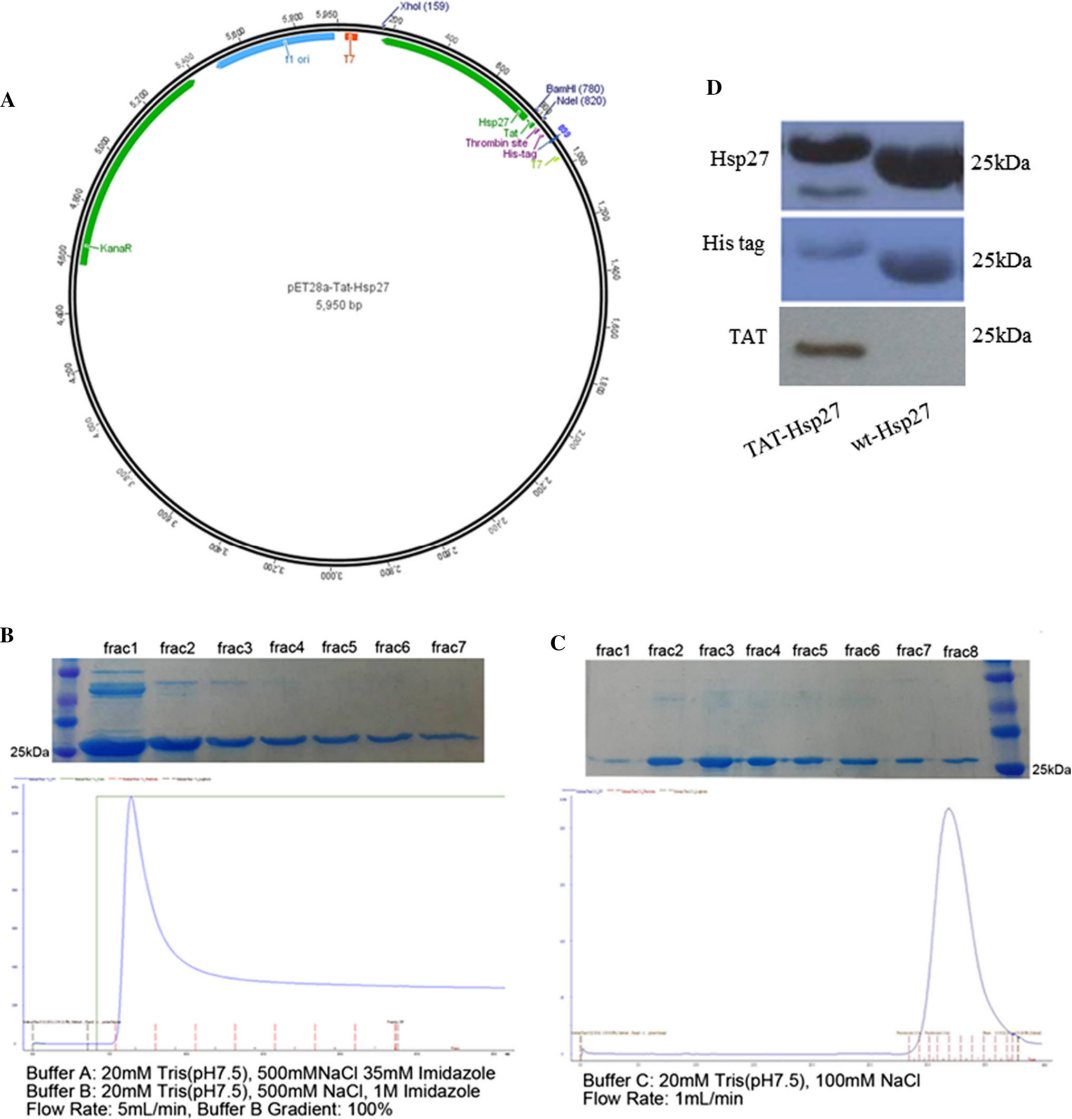
Purification of His-tagged Tat-Hsp27. **a** pET28a vector containing hexahistidine (His6) and Tat protein transduction domain and Hsp27 cDNA. **b** His-tagged Tat-Hsp27 was eluted from the affinity chromatography column using isocratic elution buffer (20 mM Tris–HCl, pH 7.5, 500 mM NaCl, and 1 M imidazole). **c** His-tagged Tat-Hsp27 was further purified using size excluion chromatography (20 mM Tris–HCl, pH 7.5, and 100 mM NaCl) and eluted as a single peak. Fractions were analyzed using SDS-PAGE after each purification step. **d** Western blot analysis of purified His-tagged Tat-Hsp27 using anti-His tag, anti-Hsp27, and anti-Tat antibodies

The sequence encoding Tat-Hsp27 fusion protein was cloned into the pET28a vector, which produces a recombinant protein with a hexahistidine tag (Fig. 1a) and purified using affinity purification and size exclusion chromatography. Fractions containing His-tagged Tat-Hsp27 were identified using 12 % SDS-PAGE analysis with Coomassie Brilliant Blue staining. His-tagged Tat-Hsp27 in fractions 1–6 exhibited high affinity for the affinity matrix. The eluted His-tagged Tat-Hsp27 was further purified using size exclusion chromatography. Fractions containing His-tagged Tat-Hsp27 were identified using 12 % SDS-PAGE with Coomassie Brilliant Blue staining. His-tagged Tat-Hsp27 eluted as a single peak from size exclusion chromatography, as confirmed by Western blot analysis (Fig. 1d), and migrated to a position slightly above the 25-kDa molecular weight marker in 12 % SDS-PAGE analysis. The purified recombinant His-tagged wt-Hsp27 and Tat-Hsp27 was concentrated to 5.85 and 1.438 mg/mL as determined using the BCA assay, respectively.

**Fig. 2:**
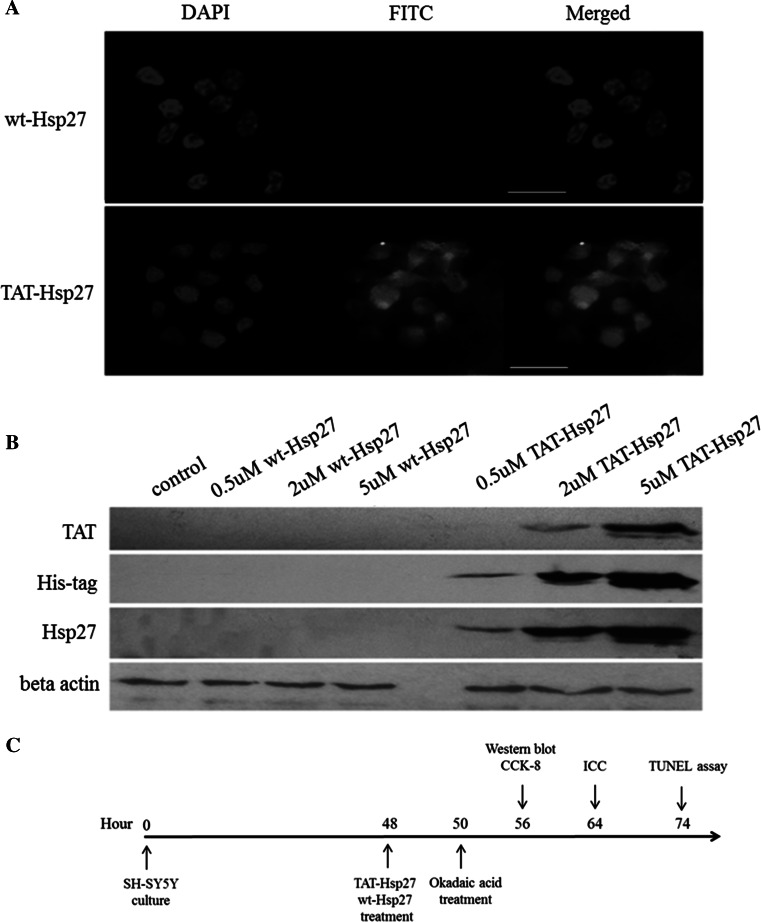
Transduction of wt-Hsp27 and Tat-Hsp27 into SH-SY5Y cells. **a** Immunocytochemistry. FITC-conjugated wt-Hsp27 or Tat-Hsp27 was delivered into cells and analyzed 2 h later. Image restoration microscopy indicates that Tat-Hsp27 was rapidly and efficiently delivered, in contrast to wt-Hsp27. *Scale bar* 30 μm. **b** Protein from cell lysates of SH-SY5Y cells treated with wt-Hsp27 or Tat-Hsp27 was analyzed using Western blot analysis. The protein expression was normalized to actin. **c** Experimental design for Tat-Hsp27 treatment in SH-SY5Y cells

To investigate whether Tat-Hsp27 could be effectively delivered into cells, we conjugated FITC to Tat-Hsp27. Image restoration microscopy indicated that FITC-Tat-Hsp27 was efficiently delivered into SH-SY5Y cells following treatment for 2 h at a concentration of 2 μM, in contrast to 2 μM wt-Hsp27. We also performed Western blot analysis to confirm the intracellular delivery of Tat-Hsp27 and to determine whether its transduction is dependent on the Tat-Hsp27 concentration. SH-SY5Y cells were treated with Tat-Hsp27 for 2 h and were lysed using RIPA buffer. As shown in Fig. 2b, Hsp27 was present in the lysate of SH-SY5Y cells treated with 2 and 5 μM Tat-Hsp27, whereas equivalent concentrations of wt-Hsp27 did not transduce cells. Additionally, increasing the concentration of Tat-Hsp27 used to treat cells resulted in an increased level of delivered protein. Therefore, the recombinant Tat-Hsp27 can be delivered into cells in a concentration-dependent manner.

**Fig. 3:**
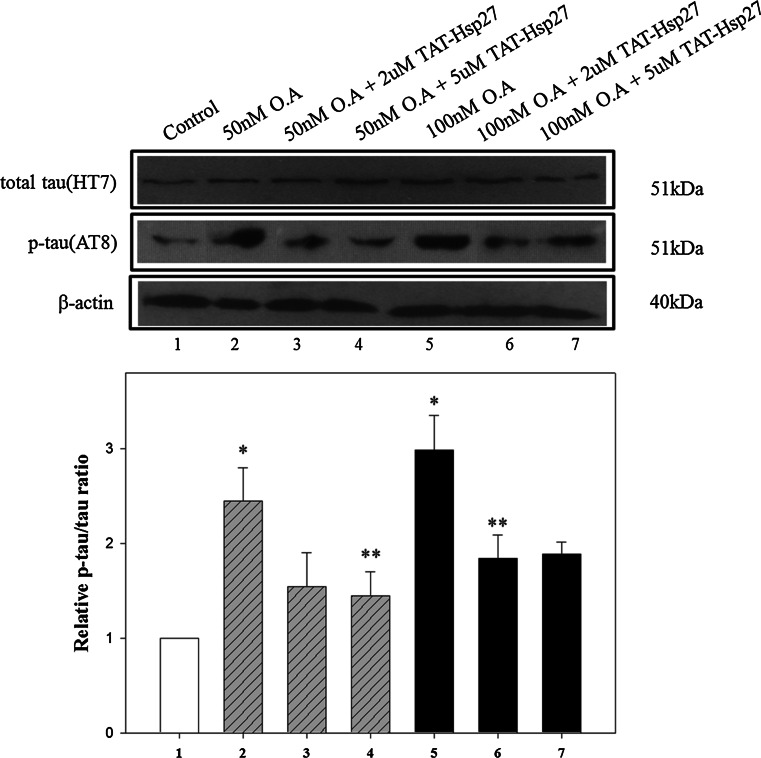
Effect of Tat-Hsp27 on tau hyperphosphorylation. Western blot analysis of total tau (HT 7) and p-tau(AT 8), with β-actin as a loading control. *p < 0.05 compared with the control. **p < 0.05 compared with okadaic acid alone. Values indicate mean ± SD (Anova, Tukey’s HSD test, *n* = 3 per group)

**Fig. 4:**
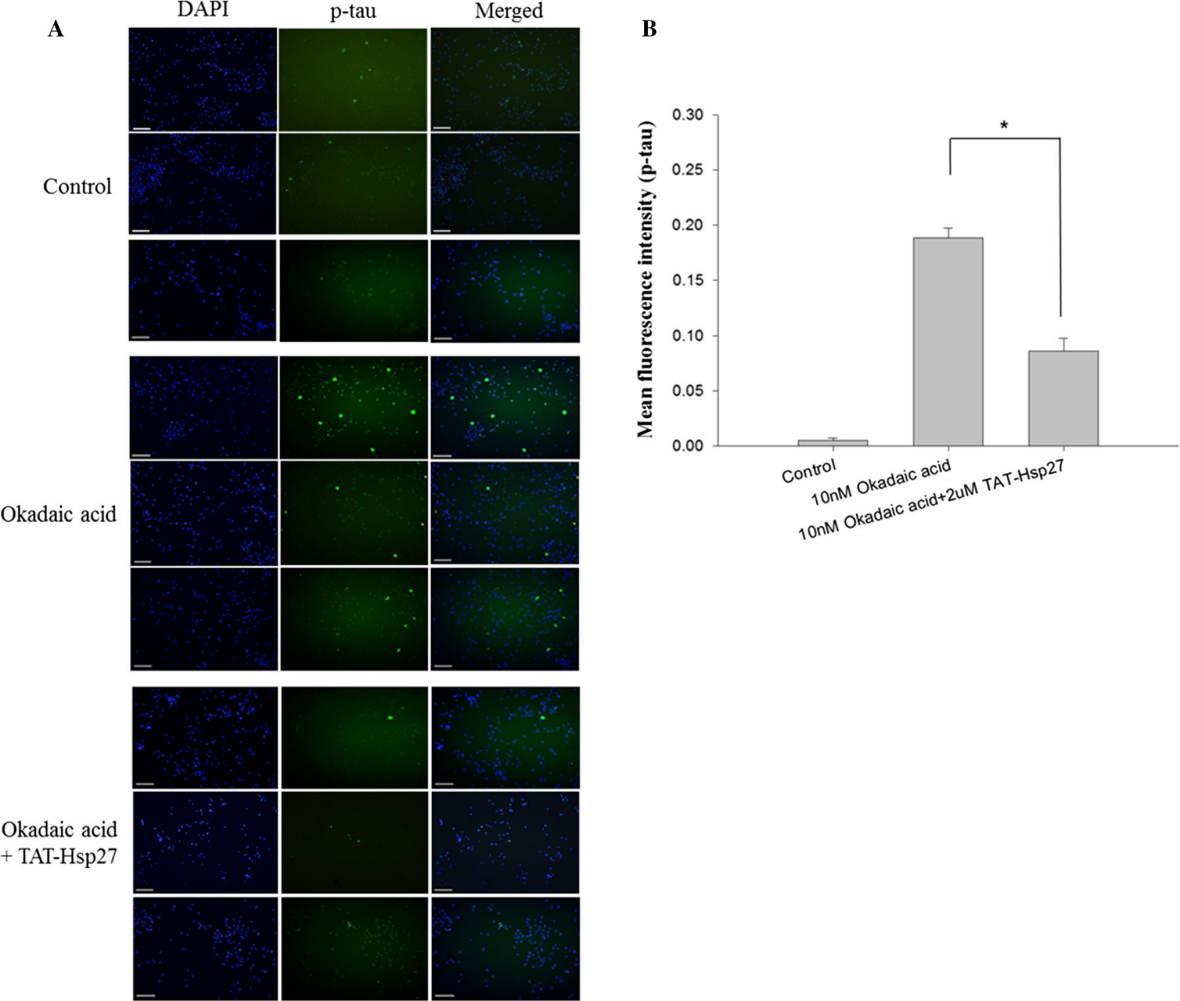
Representative immunofluorescence images. **a** SH-SY5Y cells were treated with 2 μM Tat-Hsp27 (2 h) followed by 10 nM okadaic acid (14 h). All cells were fixed and stained with primary antibodies against p-tau(AT 8) and the corresponding fluorescent secondary antibody. Green (FITC) indicates phosphorylated tau. *Scale bar* 100 μm. **b** Quantitation of the immunofluorescence intensity demonstrated that phosphorylated tau by Okadaic acid was decreased by treatment with Tat-Hsp27. Values indicate mean ± SD (Anova, Tukey’s HSD test, *n* = 3 per group). *p < 0.05 compared with okadaic acid alone

Because Hsp27 is known to have an effect on phosphorylation, we investigated whether Hsp27 can reduce the level of hyperphosphorylated tau, which is implicated in the pathogenesis of AD. SH-SY5Y cells were treated with 2 or 5 μM Tat-Hsp27 for 2 h, and the phosphorylation of normal tau was induced by the addition of 50 or 100 nM okadaic acid. To quantify the relative phosphorylation, we normalized the level of hyperphosphorylated tau (p-tau) in Western blot analysis to the level of total tau using SigmaPlot software. Following treatment with 50 or 100 nM okadaic acid for 2 h, the level of hyperphosphorylated tau was approximately 2.4-fold or three-fold greater than the control group, respectively. Following treatment with 50 nM okadaic acid, the level of p-tau in cells pretreated with 2 and 5 μM Tat-Hsp27 decreased with an increasing concentration of Tat-Hsp27 (Fig. 3), whereas equivalent concentration of tat peptide did not prevent tau hyperphosphorylation and oligomers (Supplementary Fig. S1). Tat-Hsp27 did not revert tau phosphorylation and aggregation into oligomers when added at the same time or after okadaic acid (as data not shown). Following treatment with 100 nM okadaic acid, cells pretreated with 5 μM Tat-Hsp27 showed slightly higher levels of p-tau than cells pretreated with 2 μM Tat-Hsp27. This finding suggest that when tau is hyperphosphorylated, the protective effect was better 2 than 5 μM Tat-Hsp27 on the reduction in hyperphosphorylated tau. Therefore, we used 2 μM Tat-Hsp27 in subsequent experiments. We also performed immunocytochemistry using an anti-p-tau antibody conjugated to FITC (Fig. 4). SH-SY5Y cells were treated with 2 μM Tat-Hsp27 for 2 h and were subsequently treated with 10 nM okadaic acid for 14 h. The level of p-tau was significantly decreased compared with treatment with okadaic acid alone (*n* = 3).

**Fig. 5:**
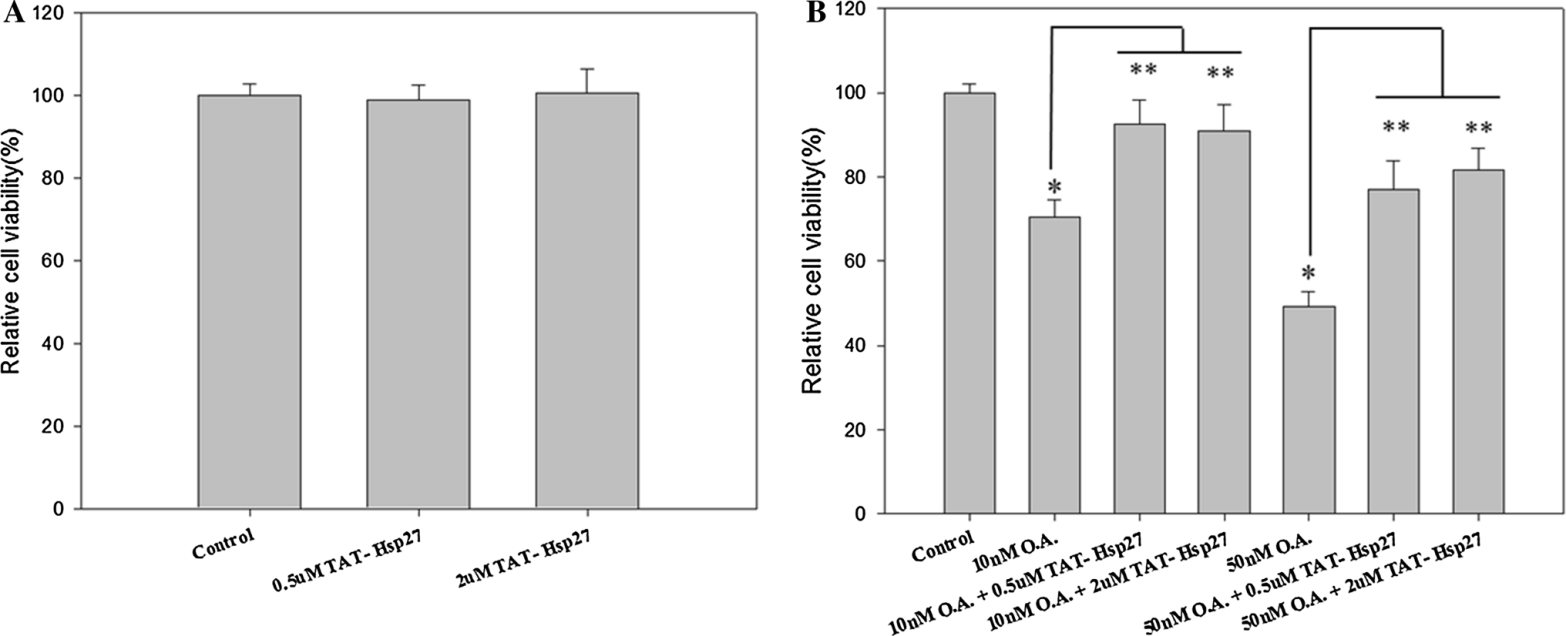
Cytotoxicity assay using the Cell Counting Kit-8. SH-SY5Y cells were transduced with 0.5 or 2 μM Tat-Hsp27 alone (**a**) or prior to treatment with 10 and 50 nM okadaic acid (**b**). The transduction of Tat-Hsp27 alone does not affect the cell viability. *p < 0.05 compared with the control. **p < 0.01 compared with okadaic acid alone. Values indicate mean ± SD (Anova, Tukey’s HSD test, *n* = 7 per group)

**Fig. 6:**
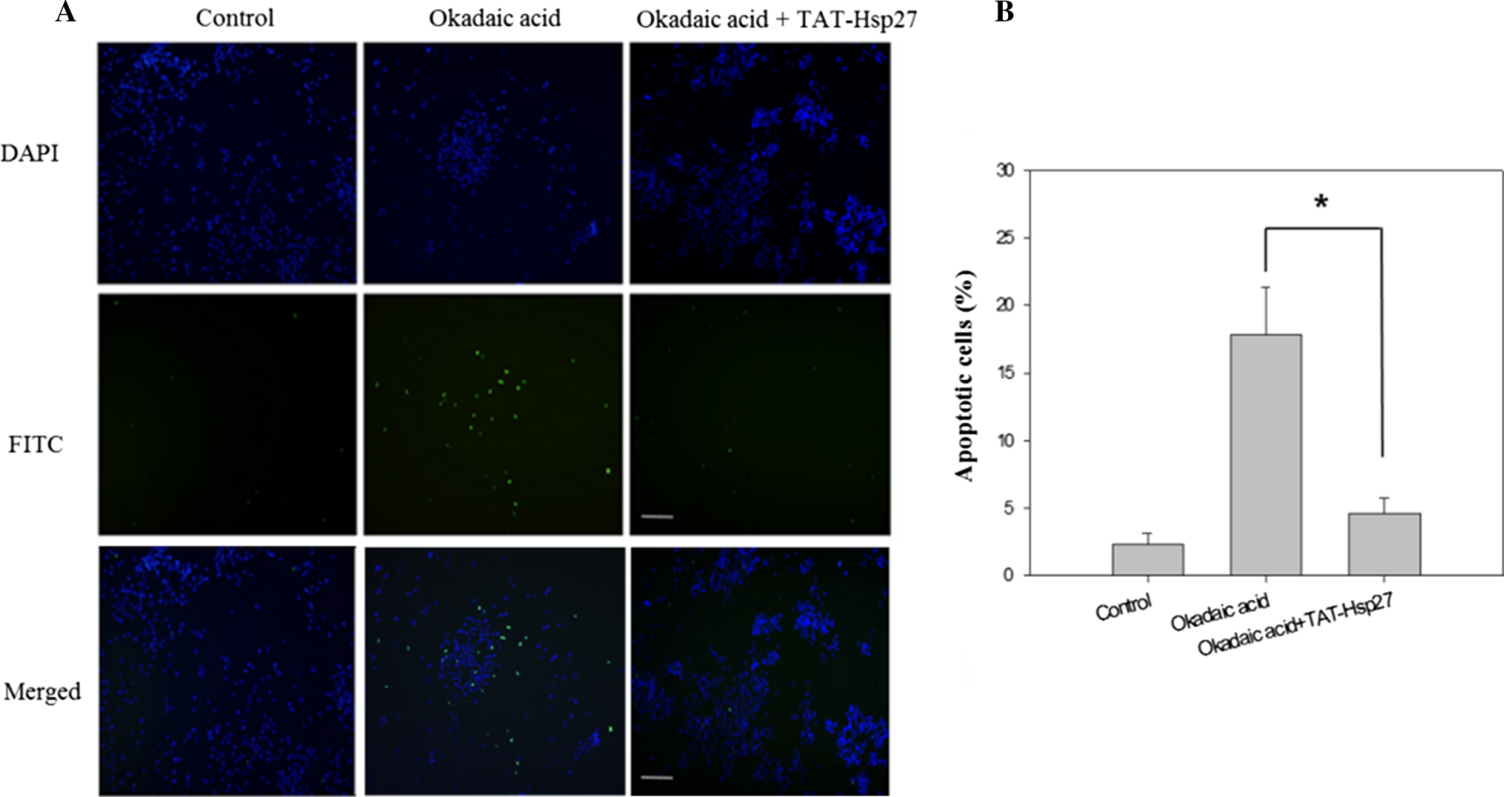
Tat-Hsp27 inhibits apoptotic cell death induced by okadaic acid. The TUNEL assay was performed to determine the extent of programmed cell death. SH-SY5Y cells were treated with 10 nM okadaic acid alone or following pretreatment with 2 μM Tat-Hsp27. **a** Representative images of the TUNEL assay. *Scale bar* 100 μm. **b** Quantitation of the percentage of TUNEL-positive cells indicated that apoptosis induced by okadaic acid was decreased in cells pretreated with Tat-Hsp27. Values indicate mean ± SD (Anova, Tukey’s HSD test, *n* = 3 per group). *p < 0.05

A cytotoxicity assay was performed to determine whether Tat-Hsp27 directly affected the cell death induced by hyperphosphorylated tau. We first evaluated the relative cell viability (RCV) in the presence of Tat-Hsp27 alone. As shown in Fig. 5a, treatment with Tat-Hsp27 alone did not alter the RCV of normal cells. In contrast, SH-SY5Y cells treated with 10 and 50 nM okadaic acid for 6 h exhibited a decrease in 70.45, 49.234 % cell viability (*p < 0.05). When cells were treated with 0.5 or 2 μM Tat-Hsp27 followed by treatment with 10 and 50 nM okadaic acid, the cell viability increased to 92.44, 90.78, 76.89, and 81.57 %, respectively (**p < 0.01; Fig. 5b). Cells treated with 0.5 and 2 μM Tat-HSP27 clearly showed a protective effect against cell death induced by hyperphosphorylated tau. Treatment with 10 nM okadaic acid alone for 14 h greatly increased the number of TUNEL-positive cells, whereas the cells pretreated with 2 μM Tat-Hsp27 exhibited decreased TUNEL positivity (Fig. 6). In the control group, apoptotic cells comprised 2.29 % of the total number of cells. Following exposure to 10 nM okadaic acid, the percentage of apoptotic cells increased to 17.84 %. However, this increase was inhibited by Tat-Hsp27 (4.56 %). Therefore, intracellular delivery of Tat-Hsp27 prevented the apoptotic cell death induced by hyperphosphorylated tau.

## Discussion

In this study, the recombinant Tat-Hsp27 reduced the level of hyperphosphorylated tau induced by okadaic acid in SH-SY5Y neuroblastoma cells and prevented the apoptosis induced by abnormal tau aggregates in our cellular model of AD. The effect of Hsp27 on phosphorylated tau has recently received increasing attention. However, few studies have examined the therapeutic effect of Hsp27 on hyperphosphorylated tau, which has been implicated in the pathogenesis of AD. We demonstrate that Hsp27 exhibits a protective effect on apoptotic cell death caused by pathological tau. This finding suggests that Hsp27 may represent a potential protein therapeutic for AD.

Tau stabilizes microtubules; however, tau mutations that result in its hyperphosphorylation lead to the formation of tau filaments that can form twisted ribbons or rope-like filaments (Kosik and Shimura 2005). Using phosphorylation-dependent monoclonal antibodies against tau, mass spectrometry, and sequencing, at least thirty phosphorylation sites have been reported (Bussiére et al. 2000). Because the phosphorylation of tau is regulated by various kinases, including proline-directed protein kinases and glycogen synthase kinase 3, and phosphatases, including Ser/Thr protein phosphatases 1, 2A, 2B (calcineurin) and 2C, we used okadaic acid, which is a protein phosphatase inhibitor, to induce PHF-like hyperphosphorylation of tau (Zhang and Simpkins 2010). Hsp27 facilitates the degradation and prevents the aggregation of aberrant substrates independent of ATP or ubiquitination (Jakob et al. 1993; Shimura et al. 2004a, b). The mechanism by which Hsp27 prevents apoptosis could be from not only the interaction between Hsp27 and the pathologically hyperphosphorylated tau but also from Hsp27-mediated inhibition of pro-caspase-9 and caspase-3 (Concannon et al. 2003). PP2A dephosphorylates Hsp27 more effectively than that of PP1, which is weakly active. Many of the sites that are dephosphorylated by PP2A are phosphorylated by either GSK-3β or Cdk5. These include S199, S202, T205, S396, and S404 93-96. We used the AT8 antibody to detect phosphorylated tau at Ser202 and Thr205 (Goedert et al. 1995). Okadaic acid inhibits Ser/Thr protein phosphatases and can induce tau hyperphosphorylation and neurodegeneration (Kamat et al. 2013).

A recent study reported that hyperphosphorylation of tau leads to a 20-fold inhibition of the tau–tubulin binding affinity. This finding supports the critical role for tau in the pathogenesis of NFT-induced degeneration because the balance between kinases and phosphatases is disturbed in AD, resulting in the disassociation of tau from microtubules and its subsequent aggregation (Wischik et al. 2014). In our previous study, we showed that hyperphosphorylated tau, but not the overexpression of normal tau alone, reduces the cell viability of the neuroblastoma cell line SH-SY5Y (Ahn et al. 2013). Thus, in the present study, a cellular model of AD was induced by the hyperphosphorylation of endogenous tau.

Moreover, we utilized the PTD of the HIV Tat protein to enhance the delivery of Hsp27, thereby enhancing its protective effect (Wadia and Dowdy 2005; Stetler et al. 2010). Conjugation with specific peptide sequences, which are termed PTDs or cell-penetrating peptides, improves the delivery of a range of agents, including antisense oligonucleotides, plasmids, microbeads, and liposomes, which suggests that these peptide sequences may represent a universal in vitro and in vivo cellular delivery system (Green et al. 2003). We confirmed that the fusion protein of Hsp27 and Tat PTD was delivered into normal human neuroblastoma SH-SY5Y cells and into SH-SY5Y cells containing hyperphosphorylated tau induced by okadaic acid treatment. To determine whether Tat-Hsp27 has a protective effect against okadaic acid-induced cell death, SH-SY5Y cells containing hyperphosphorylated tau were treated with the fusion protein. The levels of phosphorylated tau significantly decreased compared with the cells that were not treated with Tat-Hsp27, and the relative cell viability was enhanced. This result suggests that Tat-Hsp27 represents a potential protein therapeutic to reduce hyperphosphorylated tau.

Protein delivery systems offer several advantages, such as the ease of production from synthetic or natural compounds which provoke low inflammatory response (Rogers and Rush 2012). Compared to other delivery systems, such as gene delivery utilizing plasmids or viral vectors, our recombinant Tat-Hsp27 has several advantages. The straightforward purification of Tat-Hsp27 is amenable to large-scale production. Moreover, Tat-Hsp27 is nontoxic and stable and can be readily delivered into cells.

As previously mentioned, PTDs can readily and rapidly deliver proteins into cells. Drugs to treat neurodegenerative diseases must be capable of penetrating the blood–brain barrier (BBB) to be effectively delivered (Ozsoy et al. 2009). However, greater than 98 % of all potential CNS drugs cannot cross the BBB (Brasnjevic et al. 2009). Hence, we designed a recombinant Tat-Hsp27 fusion protein to treat a cellular AD model, and this fusion protein was delivered into cells at high levels within 2 h (Fig. 2). Therefore, we anticipate that the uptake of Tat-Hsp27 into the brain will readily occur when nasally administered and will protect against cell death caused by hyperphosphorylated tau, which is one of the primary causes of AD (Brasnjevic et al. 2009; Schwarze et al. 1999). Our results suggest that Tat-Hsp27 has a protective effect against hyperphosphorylated tau and could represent a valuable protein therapeutic for AD.

## Electronic supplementary material

Below is the link to the electronic supplementary material.

Tat-Hsp27 pretreatment ameliorates tau hyperphosphorylation and aggregation. Protein from cell lysates of SH-SY5Y cells treated with Tat-Hsp27 or Tat peptide was analyzed using Western blot analysis with p-tau(AT 8), β-actin. Arrow was presented tau oligomers. (DOCX 112 kb)
