# Supplementary figures and images for: Protective Effect of Tat PTD-Hsp27 Fusion Protein on Tau Hyperphosphorylation Induced by Okadaic Acid in the Human Neuroblastoma Cell Line SH-SY5Y
Source: Cell Mol Neurobiol. 2015 May 20;35(7):1049–59. doi: 10.1007/s10571-015-0199-1 (PMC4572059; doi:10.1007/s10571-015-0199-1)

Supplementary Figure S1

**
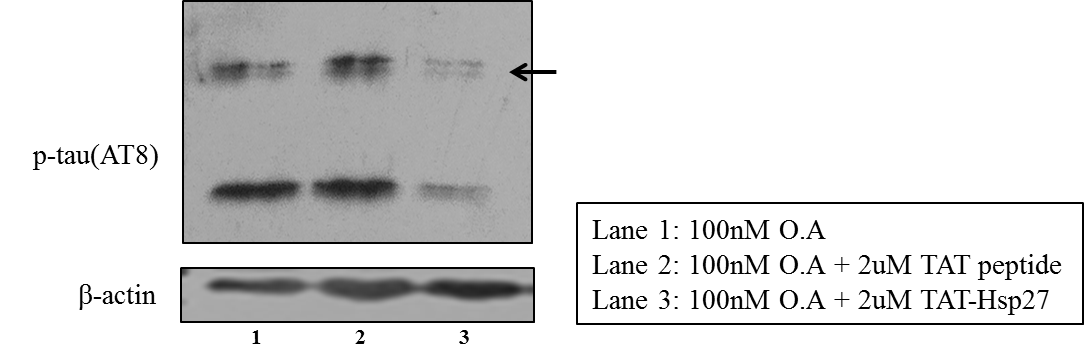
**

Supplement: Supplementary file 1 — Tat-Hsp27 pretreatment ameliorates tau hyperphosphorylation and aggregation. Protein from cell lysates of SH-SY5Y cells treated with Tat-Hsp27 or Tat peptide was analyzed using Western blot analysis with p-tau(AT 8), β-actin. Arrow was presented tau oligomers. (DOCX 112 kb) [file 10571_2015_199_MOESM1_ESM.docx]
